# Dr. John Orlando Roe

**Published:** 1916-02

**Authors:** 


**Dr. John Orlando Roe,** P. & S. 1871, died at his home in Rochester Dec. 24, 1915, of anaemia. He was born in 1848 in Patchogue, L. I. He practiced in Rochester for about 40 years, devoting his attention to laryngology and specializing in plastic and cosmetic operations upon the nose and face. It may almost be said that he created this special department of laryngology in the broader sense. At least, he had done a large amount of original work in it and had devised special instruments for carrying out the principles of operation which he conceived. Besides his affiliation with the regular organizations of the profession and local societies, he was a past-president of the American Medical Assn., the American Laryngological Assn. and the Medical Society of the State of N. Y. He was also a fellow of the following: American Climatological Assn., American College of Surgeons, Pan-American Medical Congress, N. Y. Academy of Medicine, Congress of American Physicians, Medical Association of Central N. Y., British Medical Assn., French Society of Otology, Laryngology and Rhinology. He was largely instrumental in founding the Rochester Academy of Medicine and in establishing the various local societies in a permanent home with a medical library. He was both a practitioner and an investigator. His special interests did not detract from his interest and activity in professional organizations for the welfare of the whole profession. Eminently successful in every sense, he was keenly sympathetic with the humblest member of his profession and beloved as a man.

